# Supplementary material for: Identification and Validation of UPF1 as a Novel Prognostic Biomarker in Renal Clear Cell Carcinoma
Source: Genes (Basel). 2022 Nov 20;13(11):2166. doi: 10.3390/genes13112166 (PMC9690017; doi:10.3390/genes13112166)
Supplement: Supplementary file 1 [file genes-13-02166-s001.zip › Figure S1-S5.pdf]

Figure S1

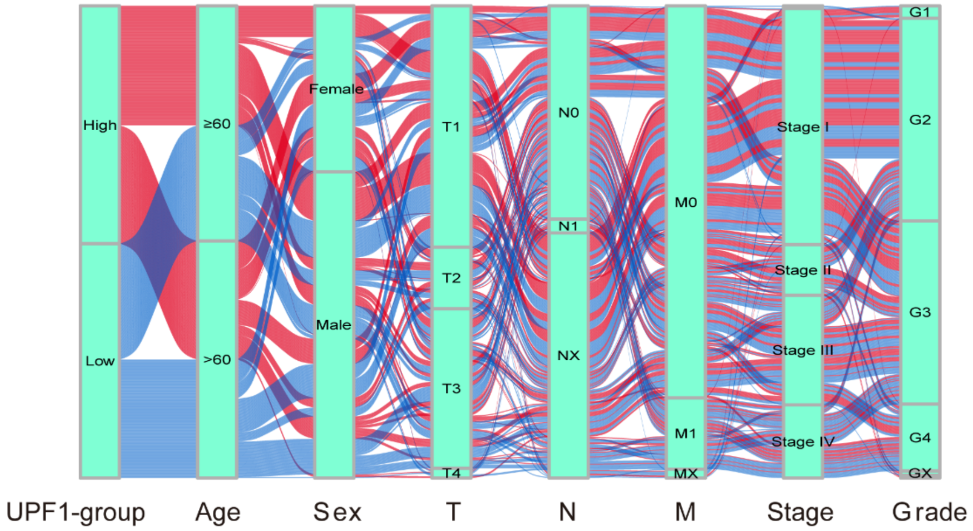

Figure S1. Alluvial plot shows the relationship between the expression of UPF1 the clinical characteristics of ccRCC patients

Figure S2

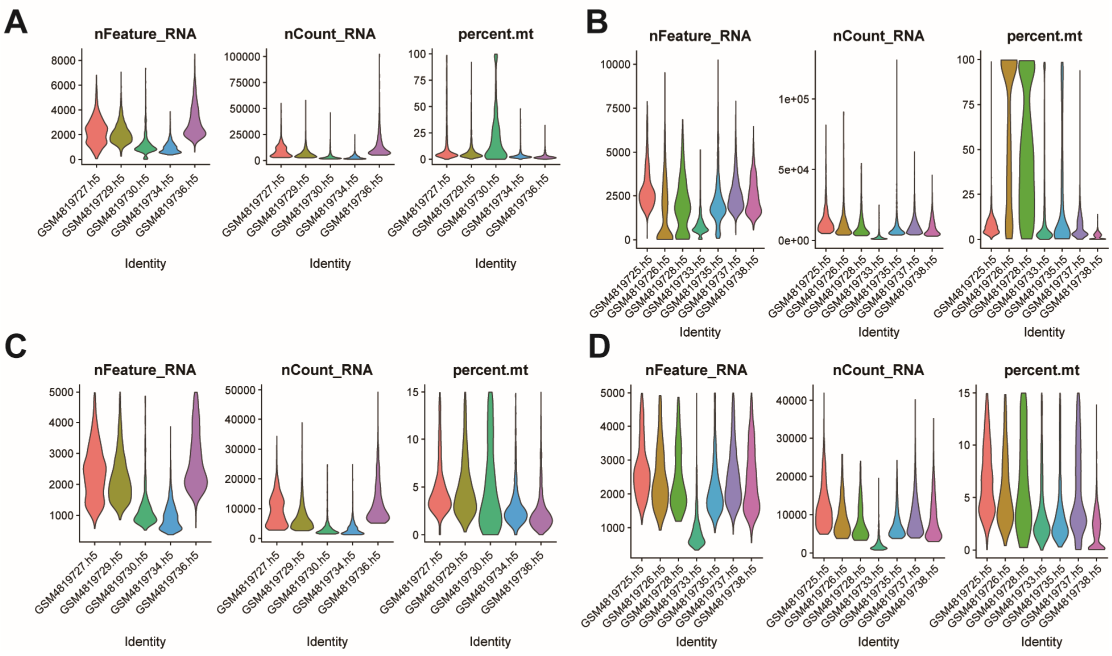

Figure S2. Normalization of single-cell sequencing data.

Figure S3

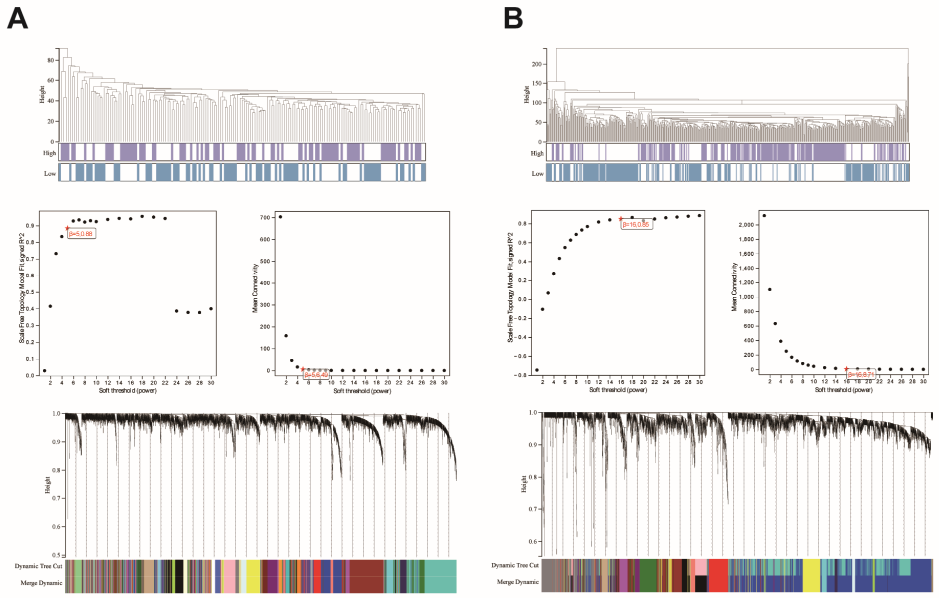

Figure S3. Construction of WGCNA network in GEO(A) and TCGA(B) cohorts

Figure S4

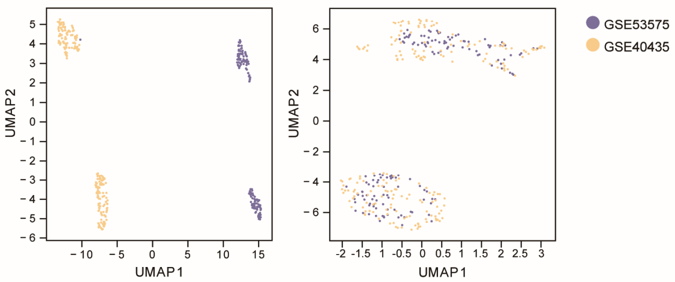

Figure S4. Batch correction of GEO datasets.

Figure S5

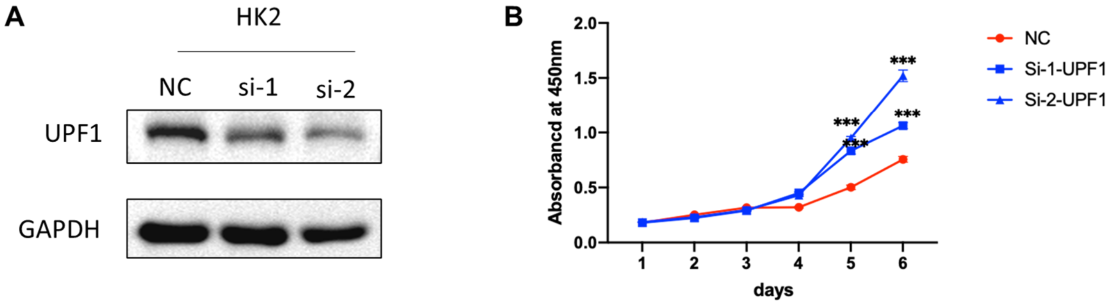

Figure S5. (**A**) knocked out UPF1 in normal renal cell lines (HK2). (**B**)The CCK8 increment curve suggested that the knockdown of UPF1 would also promote the proliferation of HK2.
